# Fibromyalgia diagnosis and biased assessment: Sex, prevalence and bias

**DOI:** 10.1371/journal.pone.0203755

**Published:** 2018-09-13

**Authors:** Frederick Wolfe, Brian Walitt, Serge Perrot, Johannes J. Rasker, Winfried Häuser

**Affiliations:** 1 National Data Bank for Rheumatic Diseases, Wichita, Kansas, United States of America; 2 University of Kansas School of Medicine, Wichita, Kansas, United States of America; 3 Georgetown University, Washington, DC, United States of America; 4 Pain Clinic, Cochin-Hôtel Dieu Hospital, Paris Descartes University, Paris, France; 5 Faculty of Behavioral Management & Social Sciences, Psychology, Health & Technology, University of Twente, Enschede, the Netherlands; 6 Department Internal Medicine 1, Klinikum Saarbrücken, Saarbrücken, Germany; 7 Department Psychosomatic Medicine and Psychotherapy, Technische Universität München, München, Germany; University of Würzburg, GERMANY

## Abstract

**Purpose:**

Multiple clinical and epidemiological studies have provided estimates of fibromyalgia prevalence and sex ratio, but different criteria sets and methodology, as well as bias, have led to widely varying (0.4%->11%) estimates of prevalence and female predominance (>90% to <61%). In general, studies have failed to distinguish Criteria based fibromyalgia (CritFM) from Clinical fibromyalgia (ClinFM). In the current study we compare CritFM with ClinFM to investigate gender and other biases in the diagnosis of fibromyalgia.

**Methods:**

We used a rheumatic disease databank and 2016 fibromyalgia criteria to study prevalence and sex ratios in a selection biased sample of 1761 referred and diagnosed fibromyalgia patients and in an unbiased sample of 4342 patients with no diagnosis with respect to fibromyalgia. We compared diagnostic and clinical variables according to gender, and we reanalyzed a German population study (GPS) (n = 2435) using revised 2016 criteria for fibromyalgia.

**Results:**

In the selection-biased sample of referred patients with fibromyalgia, more than 90% were women. However, when an unselected sample of rheumatoid arthritis (RA) patients was studied for the presence of fibromyalgia, women represented 58.7% of fibromyalgia cases. Women had slightly more symptoms than men, including generalized pain (36.8% vs. 32.4%), count of 37 symptoms (4.7 vs. 3.7) and mean polysymptomatic distress scores (10.2 vs. 8.2). We also found a linear relation between the probability of being females and fibromyalgia and fibromyalgia severity. Women in the GPS represented 59.2% of cases.

**Discussion:**

The perception of fibromyalgia as almost exclusively (≥90%) a women’s disorder is not supported by data in unbiased studies. Using validated self-report criteria and unbiased selection, the female proportion of fibromyalgia cases was ≤60% in the unbiased studies, and the observed CritFM prevalence of fibromyalgia in the GPS was ~2%. ClinFM is the public face of fibromyalgia, but is severely affected by selection and confirmation bias in the clinic and publications, underestimating men with fibromyalgia and overestimating women. We recommend the use of 2016 fibromyalgia criteria for clinical diagnosis and epidemiology because of its updated scoring and generalized pain requirement. Fibromyalgia and generalized pain positivity, widespread pain (WPI), symptom severity scale (SSS) and polysymptomatic distress (PSD) scale should always be reported.

## Introduction

Beginning in1980, a series of criteria for fibromyalgia diagnosis became available [1–3], culminating in the American College of Rheumatology (ACR) 2010 preliminary criteria [4] and its subsequent self-report modifications–2011 (also known as modified 2010) [5] and 2016 [2010+][6]. These criteria sets had different perspectives and often did not identify the same patients [7]. In addition, the specific application of criteria to clinical diagnosis was and is uncommon [8]. Following the publication of the 2010+ criteria the idea that fibromyalgia could only be understood as a dichotomous entity was vitiated, as the 2010+ criteria contained a continuous measure of fibromyalgia symptom severity (fibromyalgianess)—the polysymptomatic distress scale (PSD). This scale provided important information linking the diagnosis of fibromyalgia to the continuum of symptom severity, where persons with sufficient severity are designated as having fibromyalgia [6]. Differences in criteria and methods of diagnosis contributed to varying estimates of fibromyalgia prevalence and sex distribution [9, 10].

The relation between fibromyalgia and gender is controversial and confusing [11–20]. The major view holds that at least 80–90% of diagnosed fibromyalgia occurs in women [11–15]. But 2 large population studies using modified ACR criteria reported that 60.8% and 60.5% of persons with fibromyalgia were women [16, 17].

There are, however, two types of fibromyalgia, Criteria (based) fibromyalgia (CritFM) and Clinical fibromyalgia (ClinFM) [21, 22], CritFM is a research diagnosis, primarily used in epidemiologic studies in which persons with fibromyalgia are identified based on assessments with validated, published criteria. Such persons can be unaware that they satisfy fibromyalgia criteria or “have” fibromyalgia. The definition of CritFM requires assessment methods that are valid, and that populations assessed are large, representative and selected and evaluated without bias. CritFM can be thought of as “true” fibromyalgia only if it is assumed that identified cases identify persons with a real disease or real disorder. This distinction is important, as many people do not believe that fibromyalgia is either a disease or disorder [23, 24], while others believe exactly the opposite.

It may be more useful, however, if we consider CritFM individuals to be just that, persons satisfying fibromyalgia criteria; and CritFM prevalence as being a measure of those satisfying criteria whether they have been clinically diagnosed with fibromyalgia or not. The percent of those with CritFM who are women varies widely, from ~90% to <61% [25], depending on investigators and criteria used. Most (~3/4) persons with CritFM have not received a physician diagnosis [26], leading pharmaceutical companies and some physicians to state that fibromyalgia remains undiagnosed in 75% of persons with the disorder [27].

By contrast, ClinFM is composed of persons with a reported clinical diagnosis of fibromyalgia. However, most (~3/4) of such patients did not satisfy 2010–2016 fibromyalgia criteria when studied post-diagnosis, and more than 90% of ClinFM patients were women in a US study [26]. Membership in this group can be influenced by biased (often self) referral [28, 29], misdiagnosis [30], physician and patient beliefs [31], varying diagnostic methods [8], confirmation and ascertainment bias [32], and societal and social pressures [23, 33]. ClinFM is the public face of fibromyalgia.

Studies done using ClinFM diagnosis are ClinFM studies, as they are inherently biased. CritFM can provide unbiased estimates of putative fibromyalgia provided the sample is representative and ascertainment is reliable and unbiased. These requirements can be difficult to satisfy. But ClinFM can never provide a valid and reliable measure of fibromyalgia prevalence. The validity, reliability and sources of bias in fibromyalgia diagnosis are important concerns, as diagnosis based on bias could lead to inappropriate labeling and care, misunderstanding of the nature of the disorder, and wrong estimates of prevalence and measures of clinical outcome.

In the report below, we studied three populations with 2016 fibromyalgia criteria in order to define the role of setting, severity, symptom prevalence and sex in diagnosis of fibromyalgia, and where possible to measure the extent and mechanisms of biased diagnosis. In two instances we studied CritFM and in one instance ClinFM. We hypothesized that fibromyalgia will always be more prevalent in women than men because symptoms and pain are common in women [34–37], and that the prevalence of fibromyalgia and the distribution of cases by sex is related to symptom severity, referral and confirmation bias, and to methods used for diagnosis.

## Materials and methods

### NDB datasets

We utilized the longitudinal research database of the National Data Bank for Rheumatic Diseases (NDB) to evaluate sex and prevalence issues. The details of the NDB and its activities have been reported previously [38, 39]. Briefly, beginning in 1998, the NDB has studied longitudinal outcomes of rheumatic diseases based on detailed self-report questionnaires completed by participants with rheumatic diseases. Participants are volunteers, recruited from the practices of US rheumatologists, who complete mailed or Internet questionnaires about their health at 6-month intervals. They are not compensated for their participation. The NDB uses an open cohort design in which patients are enrolled continuously. Beginning in 2010 the NDB added fibromyalgia criteria items to its semiannual research questionnaire. This onset date was consistent with the then new American College of Rheumatology (ACR) 2010 preliminary criteria for the diagnosis of fibromyalgia (ACR 2010) [4]. In this study we created 2 datasets from the NDB database by placing adult patients with observations beginning in 2010 into datasets according to referral diagnosis. Except for diagnosis, the content of the 2 datasets was the same. The first dataset (S1 File) contained a 100% sample of 1,761 participants referred to the NDB with a diagnosis of fibromyalgia. None of these patients had rheumatoid arthritis (RA) or any other inflammatory disease. We selected this data set to describe the sex distribution of patients referred to the NDB by physicians. This dataset was representative of biased selection because referring physicians had to decide which patients had fibromyalgia and which to refer. Where patients had multiple semi-annual questionnaire assessments in the data set, we randomly selected a single questionnaire observation for study using Stata software [40].

We used a longitudinal dataset of RA patients (S2 File) to study sex, prevalence and severity among persons with fibromyalgia. The RA patients referred to the NDB were referred only because they had RA. They had not been evaluated in any way for the presence of fibromyalgia and were not selected in any way for fibromyalgia characteristics. Thus, this dataset was unbiased with respect to fibromyalgia diagnosis. As with S1 File, only 1 observation per patient was required for analysis. Using randomization software from Stata [40], we selected a random observation from the 100% sample of 12,037 RA participants (9,866 women and 2,171 men). To make analysis simpler and easier for readers to understand, we balanced the sex distribution by randomly selecting 2,171 women from the 9,866 women, and we combined the 2,171 woman and 2,171 men into a single dataset for further study. The purpose of this dataset was to assess fibromyalgia diagnosis and symptoms in patients whose selection was unrelated to fibromyalgia status. The definition of a positive fibromyalgia case among the RA patients was conditioned on satisfying the 2016 revision of the ACR 2010 criteria [6].

### NDB fibromyalgia and clinical variables [4, 6]

WPI (0–19): The widespread pain index is a summary count of the number of 19 painful regions from the Regional Pain Scale (RPS), a self-reported list of painful regions [41].

SSS (0–12): The Symptom severity scale is the sum of the severity scores of 3 symptoms (fatigue, waking unrefreshed, and cognitive symptoms) (0–9) plus the sum (0–3) of the number of the following symptoms the patient has been bothered by that occurred during the previous 6 months: (1) Headaches (0–1), (2) Pain or cramps in lower abdomen (0–1) and (3) depression (0–1).

PSD (0–31): the polysymptomatic distress scale (also known as the Fibromyalgia Severity score (FS)), is the sum of the WPI and SSS. The PSD measures the magnitude and severity of fibromyalgia symptoms in those satisfying and not satisfying criteria.

WP or widespread pain (binary variable). The WP criterion was first described in the 1990 fibromyalgia criteria [2]. Pain is considered widespread when all of the following are present: pain in the left side of the body, pain in the right side of the body, pain above the waist, and pain below the waist. In addition, axial skeletal pain (cervical spine or anterior chest or thoracic spine or low back) must be present. In this definition, shoulder and buttock pain is considered as pain for each involved side. ‘Low back’ pain is considered lower segment pain [2]. As noted elsewhere, the 1990 definition, however, is inexact because it does not state which body areas should be included in the body pain assessment. In addition, rare patients who otherwise met the 1990 criteria could satisfy the ACR widespread pain definition with pain in only a few areas. For example, in the presence of axial pain, low back pain and pain in the right hand and left foot would qualify as widespread pain. This occurs because pain in a single site can be interpreted to include more than 1 region, as when right hand pain is scored for right side and for upper extremity [42].

GP or generalized pain is a binary variable that was added to the 2016 criteria revision: pain in at least 4 of 5 body regions (left and right upper, left and right lower, axial), must be present. Jaw, chest, and abdominal pain are not included in generalized pain definition [6].

2016 criteria: A patient is considered to have fibromyalgia (satisfies modified 2016 fibromyalgia criteria) if the following 3 conditions are met:

(1) Widespread pain index (WPI) ≥ 7 and symptom severity scale (SSS) score ≥ 5 OR WPI of 4–6 and SSS score ≥ 9. (2) Generalized pain, defined as pain in at least 4 of 5 regions, must be present. Jaw, chest, and abdominal pain are not included in generalized pain definition. (3) Symptoms have been generally present for at least 3 months. (4) A diagnosis of fibromyalgia is valid irrespective of other diagnoses. A diagnosis of fibromyalgia does not exclude the presence of other clinically important illnesses [6].

#### Other study variables

Patients completed a series of questions related to the presence or absence of symptoms in the last 6 months. Selected symptoms are shown in Table 1. In addition, participants completed visual analog scales that were scored as 0–10. The scale questions and anchors were 1) severity of pain over the last week, with anchors from no pain to severe pain; 2) global severity “… all of the ways you illness affects you … rate how you are doing, with anchors of very well and very poor.” Patients reported functional status using the Health Assessment Questionnaire (HAQ) [43]. We also calculated the physical and mental component summary scores (PCS, MCS) from the Short-form 36 (SF-36) [44]. Lower values represent worse health in SF-36 variables.

**Table 1:** Probabilities, symptoms and symptom score related to fibromyalgia according to gender among RA patients in the National Data Bank for Rheumatic Diseases (S2 File).

	Females: Probability (%) or Mean (S.E.)	Males: Probability (%) or Mean (S.E.)	Differences in Probability (%) or Mean (CI)	Cases: % Female
Number of participants	2,171	2,171		
Diagnostic variables				
Fibromyalgia %	22.1%	15.6%	6.5% (4.2, 8.9%)	58.7%
Generalized pain %	36.8%	32.4%	4.4% (1.5, 7.3%)	53.2%
WPI (0–19)	5.9	4.9	1.0 (0.7, 1.3)	
SSS (0–12)	4.3	3.4	0.9 (0.7, 1.1)	
Depression %	23.6	19.0	4.6 (2.2, 7.1%)	
Headache %	29.9	17.6	11.3 (8.8, 13.8%)	
Abdominal pain %	16.2	9.4	6.8 (4.8, 8.8%)	
PSD (0–31)	10.2 (1.6)	8.2 (1.6)	2.0 (2.2)	
Symptom variables				
Dry eyes %	46.6	30.1	16.5 (13.6, 19.4%)	
Bruising %	42.6	30.4	12.2 (9.3, 15.1%)	
Dry mouth %	37.3	25.2	12.1 (9.3, 14.9%)	
Paresthesias %	46.2	37.3	8.9 (5.911.8%)	
Epigastric pain %	19.1	10.3	8.8(6.7, 10.9%)	
Nausea %	18.5	10.3	8.2 (6.1, 10.3%)	
Heartburn %	26.5	19.0	7.5 (5.0, 10.0%)	
Dizziness %	21.9	14.8	7.1 (5.0, 9.5%)	
“Nerves” %	23.2	16.2	7.0, (4.7, 9.4%)	
Oral Ulcers %	14.0	7.8	6.0 (4.2, 7.9)	
Reynaud’s	11.3	5.9	5.5 (3.8, 7.2%)	
Diarrhea %	16.6	12.2	4.9 (2.5, 6.7%)	
Vision problems %	29.4	25.4	4.0 (1.4, 6.7%)	
Photo-sensitivity %	11.8	9.3	2.6 (0.6, 4.4%)	
Hives %	4.6	2.6	2.0 (0.9, 3.2%)	
Rash %	9.7	8.1	1.6 (0.1, 3.0%)	
Dyspnea %	16.5	14.0	0.2 (-1.0, 3.8%)	
Pleurisy %	5.7	5.7	0.0 (-1.3, 1.5%)	
Asthma %	14.3	18.9	-1.4 (-3.6, 0.7%)	
Tinnitus %	23.8	31.4	-7.6 (-10.4, -4.9%)	
Hearing problem %	20.8	31.0	-10.2 (-12.9, -7.7%)	
Total symptom count	4.7	3.7	1.0 (0.8, 1.2)	
Clinical scores				
VAS Pain (0–10)	3.9	3.4	0.4 (0.3, 0.6)	
Patient global (0–10)	3.8	3.5	0.3 (0.1, 0.4)	
VAS Fatigue (0–10)	4.3	3.4	0.9 (0.7, 1.0)	
HAQ disability (0–3)	1.1	0.7	0.4 (0.3, 0.4)	
SF-36 MCS	48.4	49.2	-0.7 (-1.4, -0.3)	
SF-36 PCS	36.4	39.6	-3.2 (-3.9, -2.5)	

WPI = Widespread pain index; SSS = Symptom severity scale; PSD = Polysymptomatic distress; VAS = Visual analog scale; HAQ = Health assessment questionnaire; SF-36 MCS = Short form 36

### German population study

In 2013 we reported the results of a fibromyalgia prevalence study in the German general population using modified (2011) ACR criteria [16]. To make the results with respect to sex and prevalence similar to the NDB studies, we reanalyzed the German data using 2016 criteria, and we report those data in Table 2 (S3 File).

**Table 2:** Probabilities and symptom score related to fibromyalgia according to gender among persons in the German general population (S3 File).

	Females: Probability (%) or Mean	Males: Probability (%) or Mean	Differences in Probability (%) or Mean (CI)	Cases: % Female
Number of participants	1,308	1,137		
Diagnosis variables				
Fibromyalgia %	2.3%	1.6%	0.7% (-0.4, 1.8%)	59.2%
Generalized pain %	16.4%	13.3%	2.7% (-0.1, 5.6%)	54.5%
WPI (0–19)	1.4	1.3	0.2 (-0.4, 0.2)	
SSS (0–12)	1.8	1.5	0.3 (0.1, 0.4)	
PSD (0–31)	3.2	2.8	0.5 (0.2, 0.7)	

WPI = Widespread pain index; SSS = Symptom severity scale; PSD = Polysymptomatic distress; CI = 95% confidence interval.

#### Ethics

German population study. All participants were informed about the study procedures and signed an informed consent form. The study was approved by the Institutional Ethics Review Board of the University of Leipzig (Az 092-12-05032012). NDB study. This study was conducted in accordance with the ethical standards of the responsible committee on human experimentation and with the Helsinki Declaration of 1975, as revised in 1983. No financial support was received for this study. The study was approved by the Via Christi IRB, Wichita, Kansas, USA.

### Statistical analyses

Data were analyzed using Stata version 15.0 [40]. The primary aim of the analyses was to describe the differences in diagnostic and symptom variables according to gender. In Table 1, for each separate binary (present/absent) diagnostic or symptom variable (column 1) we used logistic regression for analysis, and for each continuous variable (PSD, WPI and SSS) we used linear regression, and we regressed these variables on sex and a quadratic component of age. As the output of the regressions (odds ratios and beta coefficients) can be difficult to interpret, we converted each odds ratio to a predicted probability and each beta coefficient to a predicted mean according to gender category using Stata’s margins procedure (columns 2 and 3). Difference data (column 4) include 95% confidence intervals. Intervals that include 0 are generally not considered to be statistically significant.

**Fig 1:**
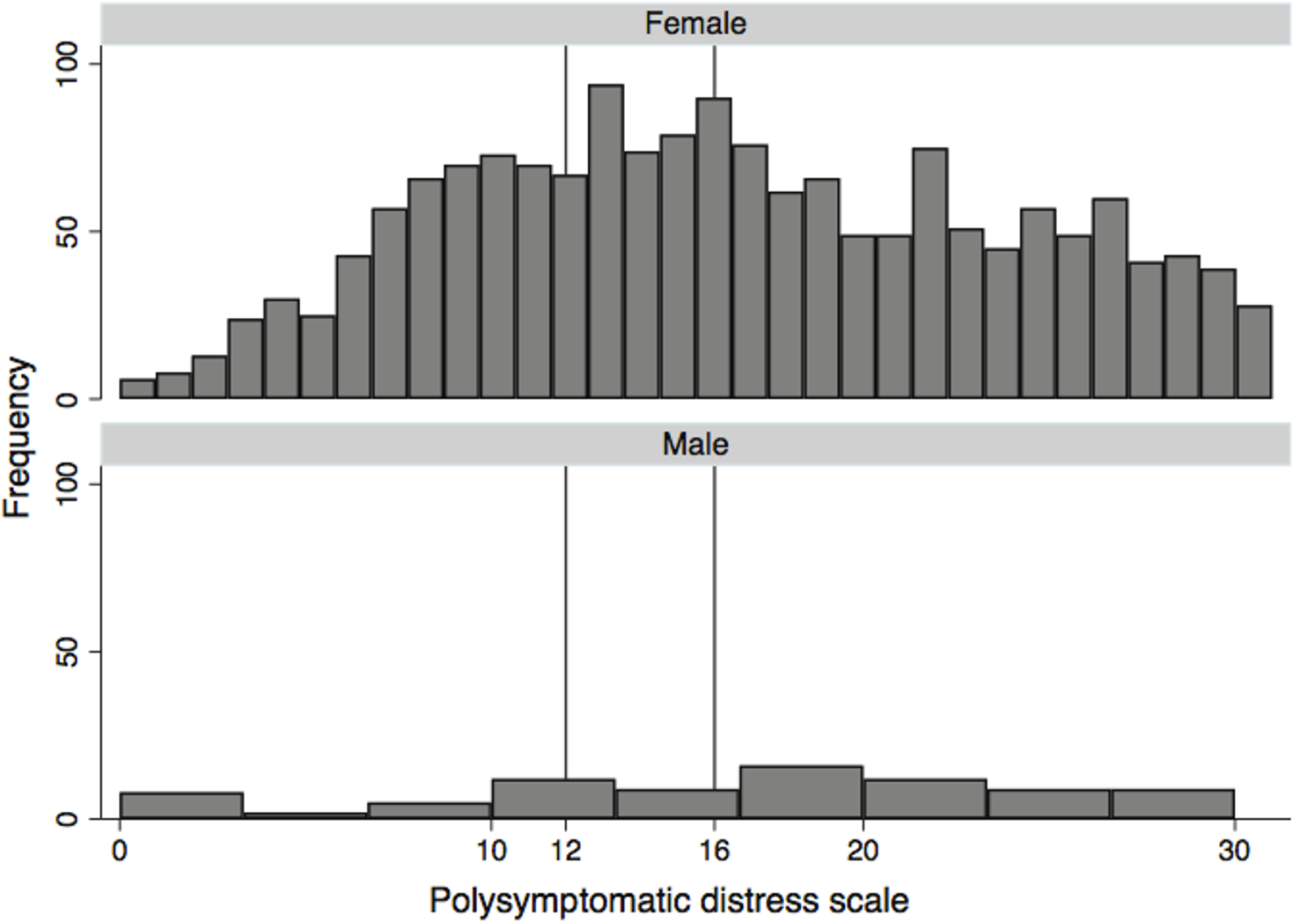
Histogram of PSD scores in patients referred to NDB with diagnosis of fibromyalgia (S1 File). A PSD ≥12 has a sensitivity of 100% for fibromyalgia diagnosis, but best cut point for correct classification is ≥16 (87.9%). Women represent 95.3% of subjects. Overall, 52.8% satisfy 2016 criteria (52.6% of women and 56.1% of men).

**Fig 2:**
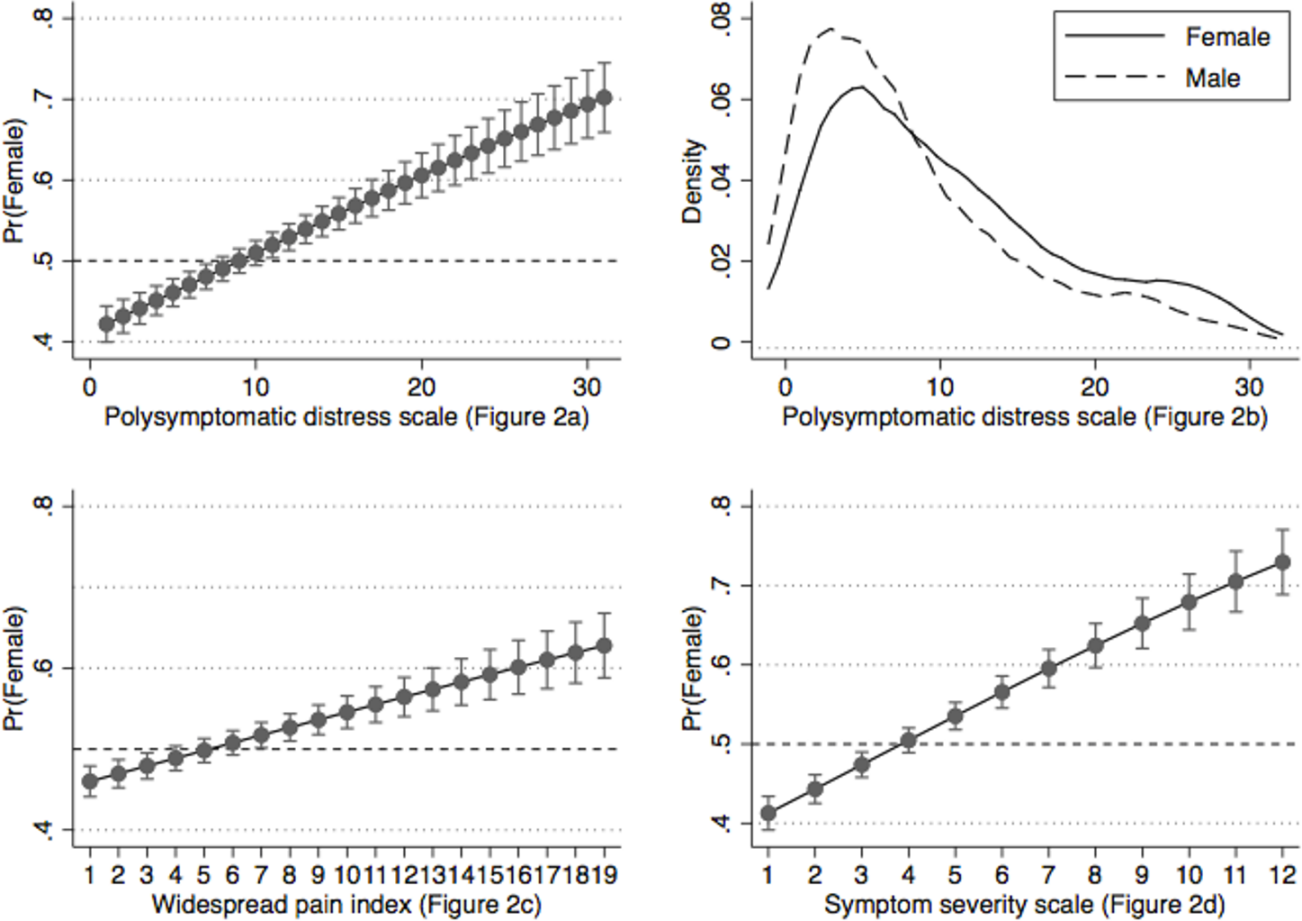
The relation of polysymptomatic distress (PSD) and its components to the sex of rheumatoid arthritis patients in the National Data Bank for Rheumatic Diseases (S2 File). (2a, 2c and 2d) The proportion of patients who are females increases with greater PSD, WPI and SSS scores. Pr = probability. (B) The distribution of PSD scores is greater and shifted to the right in women compared with men.

**Fig 3:**
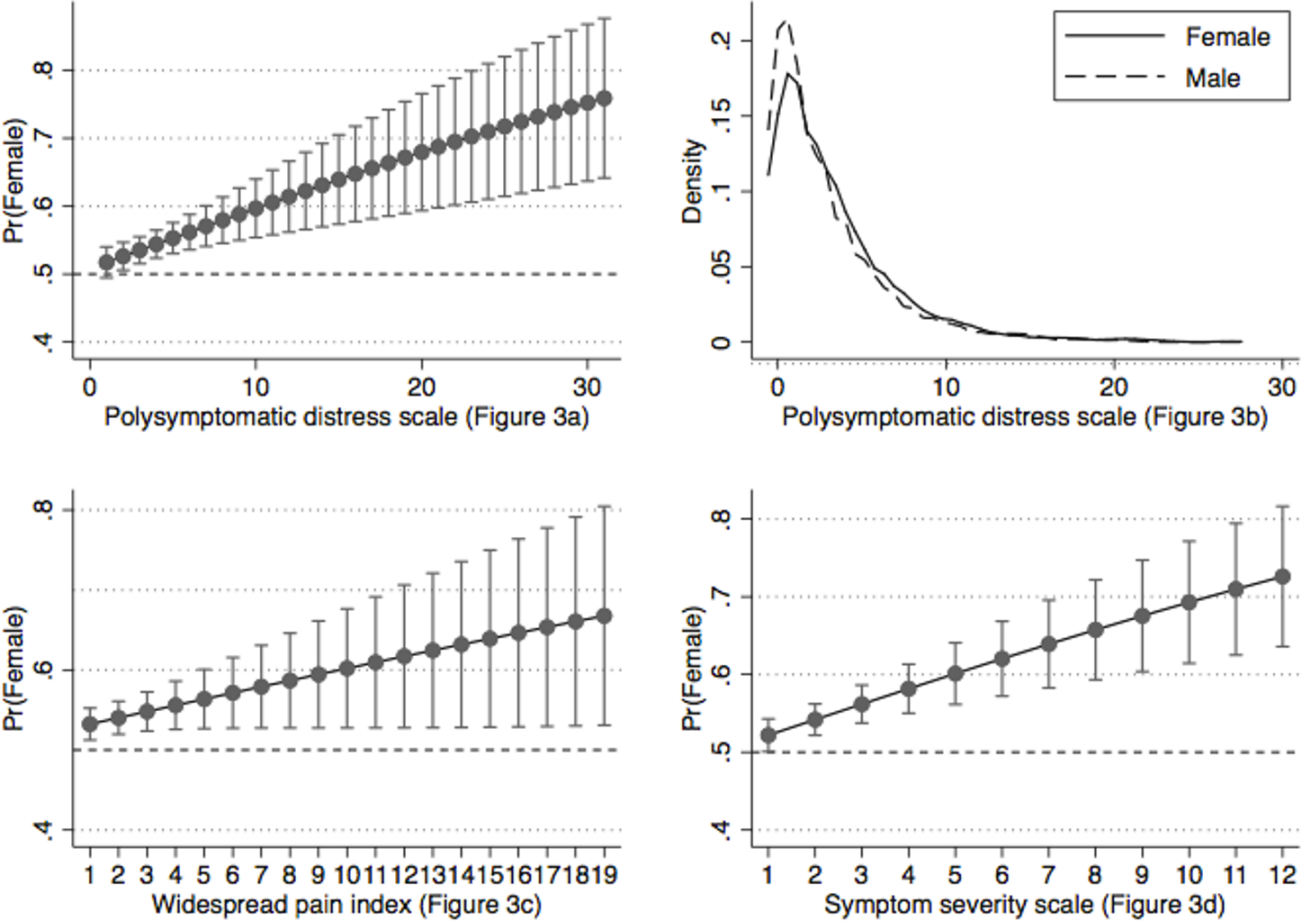
The relation of polysymptomatic distress (PSD) and its components to the sex of rheumatoid arthritis patients in the German general population (S3 File). (2a, 2c and 2d) The proportion of patients who are females increases with greater PSD, WPI and SSS scores. Pr = probability. (B) The distribution of PSD scores is greater and shifted to the right in women compared with men.

Fig 1 is a distributional histogram of PSD by sex. Non-distributional graphs in Figs 2 and 3 are representations of the relation between WPI, SSS and PSD and the probability being female at all levels of WPI, SSS and PSD as estimated from linear regression followed by Stata’s margins procedure.

Data from the German population study use the same regression and margins analysis described above to recalculate results for 2016 fibromyalgia criteria that were originally published using 2011 fibromyalgia criteria [16]. These results are not adjusted for age or any other variable.

## Results

### Biased referral fibromyalgia group (S1 File)

Fig 1 displays a histogram of PSD scores in 1761 patients referred to the NDB with a diagnosis of fibromyalgia. The PSD is strongly related to fibromyalgia diagnosis, and the area under the receiver operating curve (ROC) for diagnosis is 0.955 (95% Confidence intervals 0.946, 0.963). A PSD score ≥12 (the minimum score that allows a fibromyalgia diagnosis) has a sensitivity of 100%, but a specificity of 60.4%, and percent correctly classified of 81.3% for satisfying 2016 criteria. By ROC analysis the level of PSD with the highest percent correct (87.9%) is PSD ≥16 (sensitivity 88.5%, specificity 87.1%). Women represent 95.3% of subjects. Overall, 52.8% satisfy 2016 criteria (52.6% of women and 56.1% of men). The mean age of participants was 56.6 (S.D, 12.6). This dependence of fibromyalgia diagnosis on generalized pain and PSD is defined in the 2016 criteria and is also seen in the RA patient group and those in the German general population (Figs 2 and 3). While Fig 1 shows selection bias by sex, and hence ClinFM, it also underscores the absence of physician or evaluator bias when the 2016 criteria (S2 and S3 Files) are used compared with potential bias that is present when a tender point examination or an ACR 2010 physician evaluation is performed. To understand the proper association of sex on symptoms and fibromyalgia diagnosis, we evaluated two additional but unbiased data sets.

### Unbiased RA subjects (S2 File)

We compared individual symptoms in 2,171 men and 2,171 women with RA (S2 File). The age (SD) of the women was 59.7 (13.5) years and was 64.9 (12.0) years for men. In general, as shown in Table 1, symptoms were somewhat more common in women than men, with a mean total symptom count of 4.7 for women and 3.7 for men: difference 1.0 (95% C.I. 0.8, 1.2). These differences extended to fibromyalgia symptom and diagnosis variables. The PSD was 10.2 in women and 8.2 in men, and a PSD cut point ≥16 for the diagnosis of fibromyalgia was 92.2% accurate (ROC = 0.974). Similar increases were found for WPI (5.9 vs. 4.9), SSS (4.3 vs. 3.4) and percent with generalized pain (36.8% vs. 32.4%). Clinical severity variables were also somewhat more abnormal in women than men: VAS pain (3.9 vs. 3.4), VAS patient global (3.8 vs. 3.5), VAS fatigue (4.3 vs. 3.4), HAQ disability (1.1 vs. 0.7), SF-36 MCS (48.4 vs. 49.2) and SF-36 PCS (36.4 vs. 39.6). Finally, more women than men satisfied the 2016 fibromyalgia criteria (22.1% vs. 15.6%), and 58.7% of persons satisfying 2016 criteria were women, as were 53.2% of all persons satisfying the generalized pain criterion. Thus, in addition to fibromyalgia diagnostic variables, the non-fibromyalgia variables in Table 1 are more abnormal in women, even without considering the presence of fibromyalgia.

Further insight in the relation of fibromyalgia diagnostic variables to the sex of participants is shown in Fig 2. The distribution of PSD scores (Fig 2B) indicates that PSD scores are somewhat higher in women than men (mean difference 2.0). Roughly, fibromyalgia diagnosis positivity begins at a PSD score of 12, and more women than men have scores ≥12 and ≥16. The other panels in the figure show the relation between actual score and sex. These findings of increased score abnormality in women extend not only to pain and pain regions, but to the symptom severity and PSD scales. Thus, regardless of criterion or score or symptom addressed, women will always have more abnormalities than men and a greater likelihood of satisfying fibromyalgia criteria.

### Unbiased German population study (S3 File)

We extended the same analyses to data from the previously published German populations study [16]. As fibromyalgia is uncommon in the general population, we expected and found a lesser degree of abnormality and fewer persons with fibromyalgia (Table 2). Of particular interest, the graphs from this study that are shown in Fig 3 look very much the same as found in the RA participants of Fig 2. That is, female gender and variable severity are linked with similar appearing slopes. The main difference between the figures is that the fewer persons with fibromyalgia in Fig 3 lead to wider confidence intervals. Importantly, the proportion of fibromyalgia cases in women in the RA group is approximately the same in active RA patients (58.7%) as in the general population (59.2%). While the underlying population of these two studies was selected without bias, conforming to the definition of CritFM, in patients referred to the NDB with a diagnosis of fibromyalgia (ClinFM) more than 95% were women.

## Discussion

The results of this study show that in a general population survey or in a sample unbiased by fibromyalgia selection by physicians, 60% or fewer subjects will be women. This finding is in contradistinction most expert reports and clinical studies [9–15], including the biased referral study of this report (S1 File). We also found that given an unbiased sample, self-report criteria, such as found in the 2011 and 2016 criteria, will provide an unbiased estimate of prevalence of fibromyalgia cases and sex distribution.

In addition, we found slightly higher values of pain and symptom severity in women compared with men (Table 1), consistent with known higher values in women’s symptoms and pain reporting that exist at the biological and social level [34–37]. Because fibromyalgia is defined on the basis of pain and symptom severity—which can be estimated from PSD scores with an accuracy of 87–93% [45], this general increase in pain and symptoms in women means that women will always have higher PSD scores and therefore more CritFM than men. The extent of the increase in fibromyalgia in women compared with men is related to the difference in symptom severity in general and in PSD scores specifically. The 3 non-distributional panels of Figs 2 and 3 demonstrate that symptom severity (and the probability of fibromyalgia) is related linearly to the probability of being female. What we did not find in our unbiased CritFM samples was 9:1 female to male fibromyalgia ratios that are widely described by expert sources [11–13]. We believe that such findings only occur in the presence of selection bias or biased ascertainment.

The strength of our study is that it includes different data sources, biased and unbiased groups, very large sample sizes, and the ability to analyze at the individual level variables that make up fibromyalgia criteria with respect to sex. In addition, our results–which include reanalysis of the methodologically high quality German population study [16], are in agreement with the other high quality population study from Japan [17]. As there are no available unselected populations of pain patients, a potential limitation of our study—as well as being a strength—is that we used patients with another disease (rheumatoid arthritis) as our unbiased group and analyzed the characteristics of patients with fibromyalgia in that group. Evidence that doing this may be a strength comes from recent data that demonstrates that fibromyalgia severity variables have the same meaning and effect regardless of source of underlying pain (RA or non-inflammatory rheumatic disorders) [46].With increasing use of the 2011 and 2016 criteria, future studies should be able to further illuminate the issue of biased referral.

As unbiased epidemiological studies show only a small increase in the female to male sex ratio (~1.5:1) as opposed to the observed ratio in clinical studies of 9:1, we believe that the over-identification of fibromyalgia in women and the consequent under-identification of men is the result of bias. The central biasing factor is the widespread belief that fibromyalgia is predominantly a women’s disorder. The evidence that such beliefs exist is very strong and follows from statements of experts, respected governmental and non-governmental organizations and textbooks [11–13]. Female predominance can be observed directly in extensive pharmaceutical company advertising that shows only women with fibromyalgia, in the membership of patient support groups and in the testimony of persons with fibromyalgia who are almost exclusively women [47, 48]. With such beliefs being widespread, it is likely that physicians may anticipate, examine and diagnose fibromyalgia more in women than men. Women are referred for evaluation of fibromyalgia more often then men, and women may be more likely to consider that they have fibromyalgia and to accept the idea that they have fibromyalgia than men.

The two key biases in fibromyalgia diagnosis and diagnostic studies are selection bias and confirmation bias [49]. When selection bias is operative, patients and study subjects become study participants because of characteristics that they have that affect the probability of assessment or diagnosis. The most important distorter of fibromyalgia rates and severity is confirmation bias. Confirmation bias is the seeking or interpreting of evidence in ways that are partial to existing beliefs, expectations, or a hypothesis in hand [50]. Physicians are more likely to think of and to diagnose fibromyalgia and other somatic syndrome disorders in women than in men, an observation supported by the literature [34], including finding the 95.3% women of the 1761 patients referred to the NDB with fibromyalgia. A physician who believes the patient has fibromyalgia may unknowingly press tender point sites more vigorously (ACR 1990 Criteria) and interpret the examination response to favor fibromyalgia during the tender point examination. The same problem regarding observer bias is present when using a physician examiner with the 2010 and 2016 criteria. It has been commonly observed that some physicians believe patients; reports while other don’t [5], but the physician is required to make judgements regarding the patient’s statements when the 2010 and 2016 physician based criteria are used. And in clinical settings where physicians use gestalt based diagnosis rather than criteria, biased assessment may be even more common. By contrast, with an unbiased sample, self-report criteria, such as found in 2011 and 2016 criteria, will provide an unbiased estimate of fibromyalgia prevalence and sex distribution. Therefore we recommend the use of the 2016 self-report criteria for CritFM and as an aid to diagnosis in ClinFM.

ClinFM or clinical fibromyalgia is composed of persons with a reported clinical diagnosis of fibromyalgia. It is the public face of fibromyalgia. Many such patients do not satisfy fibromyalgia criteria when studied post-diagnosis, and membership in this group may be strongly influenced by bias. There are certain consequences of biased diagnosis that require comment. If women are over-diagnosed with fibromyalgia and men are under-diagnosed, then statistics relating to symptoms, prevalence, costs, comorbidity and clinical outcomes will be inaccurate. ClinFM can never provide valid and reliable measures of such outcomes. Our observations also suggest that there is an important element of social construction in our thinking about fibromyalgia and in its identification. While there is no easy way around this problem, as fibromyalgia has both medical and social dimensions, use of—or at least awareness of—published criteria can influence gestalt diagnosis and make physicians and researchers aware of the limitations and boundaries of diagnosis. Although it is unlikely that general physicians will use the PSD, its contemporary (not historical) use in research studies would help to advance the science of fibromyalgia by providing data on the severity of persons labeled as having fibromyalgia.

## Supporting information

S1 File This is the Dataset in Stata format for Fig 1. (DTA)

S2 File This is the Dataset in Stata format for Fig 2 and Table 1. (DTA)

S3 File This is the Dataset in Stata format for Fig 3 and Table 2. (DTA)
